# Facility-based disease surveillance and Bayesian hierarchical modeling to estimate endemic typhoid fever incidence, Kilimanjaro Region, Tanzania, 2007–2018

**DOI:** 10.1371/journal.pntd.0010516

**Published:** 2022-07-05

**Authors:** Elena R. Cutting, Ryan A. Simmons, Deng B. Madut, Michael J. Maze, Nathaniel H. Kalengo, Manuela Carugati, Ronald M. Mbwasi, Kajiru G. Kilonzo, Furaha Lyamuya, Annette Marandu, Calvin Mosha, Wilbrod Saganda, Bingileki F. Lwezaula, Julian T. Hertz, Anne B. Morrissey, Elizabeth L. Turner, Blandina T. Mmbaga, Grace D. Kinabo, Venance P. Maro, John A. Crump, Matthew P. Rubach

**Affiliations:** 1 Duke University School of Medicine, Division of Infectious Diseases and International Health, Durham, North Carolina, United States of America; 2 Duke Global Health Institute, Durham, North Carolina, United States of America; 3 Duke University School of Medicine, Department of Biostatistics & Bioinformatics, Durham, North Carolina, United States of America; 4 University of Otago, Christchurch, New Zealand; 5 Centre for International Health, University of Otago, Dunedin, New Zealand; 6 Kilimanjaro Christian Medical Centre, Moshi, Tanzania; 7 Kilimanjaro Christian Medical University College, Moshi, Tanzania; 8 Mawenzi Regional Referral Hospital, Moshi, Tanzania; 9 Kilimanjaro Clinical Research Institute, Moshi, Tanzania; Mahidol University, THAILAND

## Abstract

Growing evidence suggests considerable variation in endemic typhoid fever incidence at some locations over time, yet few settings have multi-year incidence estimates to inform typhoid control measures. We sought to describe a decade of typhoid fever incidence in the Kilimanjaro Region of Tanzania. Cases of blood culture confirmed typhoid were identified among febrile patients at two sentinel hospitals during three study periods: 2007–08, 2011–14, and 2016–18. To account for under-ascertainment at sentinel facilities, we derived adjustment multipliers from healthcare utilization surveys done in the hospital catchment area. Incidence estimates and credible intervals (CrI) were derived using a Bayesian hierarchical incidence model that incorporated uncertainty of our observed typhoid fever prevalence, of healthcare seeking adjustment multipliers, and of blood culture diagnostic sensitivity. Among 3,556 total participants, 50 typhoid fever cases were identified. Of typhoid cases, 26 (52%) were male and the median (range) age was 22 (<1–60) years; 4 (8%) were aged <5 years and 10 (20%) were aged 5 to 14 years. Annual typhoid fever incidence was estimated as 61.5 (95% CrI 14.9–181.9), 6.5 (95% CrI 1.4–20.4), and 4.0 (95% CrI 0.6–13.9) per 100,000 persons in 2007–08, 2011–14, and 2016–18, respectively. There were no deaths among typhoid cases. We estimated moderate typhoid incidence (≥10 per 100 000) in 2007–08 and low (<10 per 100 000) incidence during later surveillance periods, but with overlapping credible intervals across study periods. Although consistent with falling typhoid incidence, we interpret this as showing substantial variation over the study periods. Given potential variation, multi-year surveillance may be warranted in locations making decisions about typhoid conjugate vaccine introduction and other control measures.

## Author summary

There is evidence that typhoid fever incidence may vary over time, but there are few longitudinal studies estimating incidence. This is especially true in Sub-Saharan Africa, where recent estimates show wide variation in incidence across different settings, but very limited longitudinal descriptions from those settings. Incidence estimates were generated using facility-based surveillance data from three study periods that was adjusted for health-seeking behavior established through healthcare utilization surveys performed in the catchment area. In addition to coupling facility-based surveillance data with healthcare utilization data, we utilized a Bayesian statistical methodology in order to estimate incidence and characterize uncertainty around the estimates. Our results demonstrate moderate typhoid incidence in 2007–08 and low incidence during 2012–14 and 2016–18, but with overlapping credible intervals across study periods. Our data are consistent with evidence that endemic typhoid may vary substantially over time. Given potential variation, multi-year surveillance may be warranted in locations making decisions about typhoid conjugate vaccine introduction and other control measures.

## Introduction

Typhoid fever is an acute infection caused by *Salmonella enterica* subspecies *enterica* serovar Typhi (*Salmonella* Typhi). Typhoid fever remains a substantial cause of morbidity and mortality in low- and middle-income countries (LMIC), with an estimated 10.9 million cases in 2017 [1]. Growing evidence suggests considerable variation in typhoid incidence in endemic countries [2]. While a recent multi-center study demonstrated considerable variability with typhoid fever incidences ranging from zero to moderate or high across settings in sub-Saharan Africa [3], few sites have longitudinal incidence estimates to inform policy makers on typhoid prevention measures. The typhoid conjugate vaccine (TCV) is a major advance in typhoid prevention as, compared to the unconjugated polysaccharide vaccines, it confers more lasting immunity and does so earlier in life [4,5]. These factors led the World Health Organization (WHO) to endorse the first TCV in 2017 for children >6 months and adults <45 years living in typhoid endemic countries [6]. In light of year-on-year variation in typhoid fever incidence, longitudinal estimates constitute a relevant and important data point for typhoid endemic countries as they weigh the benefit of TCV introduction compared to prevention priorities for other diseases.

Given the utility of multiple year data in Tanzania, we sought to produce a comprehensive description of typhoid fever longitudinally across three febrile illness surveillance periods from 2007–2018 in the Kilimanjaro Region, an area previously found to have moderate to high typhoid incidence [3]. We used an extension of the multiplier method based on a Bayesian modeling approach. The multiplier method, also called ‘hybrid surveillance,’ is a well described and widely used epidemiological approach that combines hospital-based surveillance studies with community-based healthcare utilization assessments to create incidence estimates using fewer resources than active, population-based surveillance [7,8]. By incorporating a Bayesian approach, uncertainty surrounding estimates is more comprehensively assessed. We generated annual incidence estimates of typhoid fever during three study periods 2007–08, 2011–14, and 2016–18. In addition, we describe clinical characteristics of typhoid fever cases and phenotypic resistance of *Salmonella* Typhi isolates across these study periods.

## Methods

### Research ethics

The three febrile surveillance studies and two HCUS were approved by the Kilimanjaro Medical College of Tumaini University, the Tanzania National Institute for Medical Research National Research Ethics Coordinating Committee, and an Institutional Review Board of the Duke University Health System.

### Study design

We employed an extension of the multiplier method based on a Bayesian modeling approach [9,10] to estimate incidence, using data from three separate hospital-based fever surveillance studies performed at Kilimanjaro Christian Medical Centre (KCMC) and Mawenzi Regional Referral Hospital (MRRH), the two major referral hospitals in the Kilimanjaro Region. Healthcare utilization surveys were performed in the catchment area for the two sentinel hospitals.

### Hospital-based fever surveillance

#### Setting

Febrile illness surveillance was conducted in Moshi, Tanzania. Moshi is the administrative center of the Kilimanjaro Region in northern Tanzania. Moshi is at an elevation of 890 meters above mean sea level and includes Moshi Urban and Moshi Rural districts with populations of approximately 200,000 and 510,000, respectively [11]. The adjacent Hai District has an estimated population of 230,000 [11]. The climate in Moshi is tropical and is characterized by a short rain season from October through December and a longer rain season from March through May [12]. Fever surveillance was conducted at the two referral hospitals in Moshi, Tanzania: KCMC with 630 beds and MRRH with 350 beds. KCMC and MRRH are located approximately 5.5 km apart by road.

#### Study population

Febrile patients at KCMC and MRRH were prospectively enrolled from 17 September 2007 through 31 August 2008 [13,14], 26 September 2011 through 31 May 2014 [3,15,16], and 6 September 2016 through 5 September 2018 [17]. For all three study periods, the sample size was predicated upon the duration of surveillance, which was determined by project funding (i.e., sample size based upon feasibility with no sample size restriction). Fever was defined for the period 2007–08 as inpatients with an oral temperature of ≥38.0°C, and for the period 2012–14 and 2016–18 as inpatients with tympanic temperature of ≥ 38.0°C or a history of fever within the previous 72 hours. All studies enrolled inpatients, but the 2011–14 study also included outpatients; eligibility for outpatients required tympanic temperature of ≥ 38.0°C. The three studies enrolled both pediatric and adult patients, but pediatric participants were enrolled only at KCMC in the 2007–08 study and only at MRRH in the 2011–14 study. The 2016–18 study included pediatric and adult enrollment at both KCMC and MRRH and was linked to the Severe Typhoid in Africa Program (SETA) [17]. Screening and enrollment took place 08:00–16:00 hours Monday through Friday. All patients on the pediatric wards (aged ≥2 months to <13 years) and adult medical wards (aged ≥13 years) were screened for eligibility within 24 hours of admission. Outpatients were screened for fever upon registration in the out-patient department, and every other febrile patient was approached for consent to participate [3]. All minors had written informed consent given by a parent or guardian, and all adult participants provided their own written informed consent.

#### Study procedures

After obtaining informed consent, a trained clinical officer or nurse took a standardized clinical history, performed physical examination, and attempted blood culture collection on all participants. Demographic information was recorded, including age, district, and village of residence. HIV infection status for the 2007–08 period was determined with rapid antibody testing and plasma HIV RNA quantitation among participants with a negative antibody test, as previously described [13,14]. HIV status was self-reported in the 2011–14 period. The 2016–18 study period used SD Bioline (Abbott Laboratories, Abbott Park, IL) HIV-1/2 3.0 tests with confirmation by a second rapid test, Trinity Unigold (Bray, Ireland). Blood culture analysis was done using the BacTAlert 3D Microbial Detection system (bioMérieux, Marcy l’Etoile, France) with the following manufacturer bottle types: the 2007–08 and 2011–14 studies employed Pediatric FAN (PF) for participants <13 years of age and Standard Aerobic (SA) bottles for adults. The 2007–08 study also assessed for bacteremic disseminated tuberculosis using blood culture methods previously described [18]. The 2016–18 study period utilized PF Plus and FA Plus bottles for pediatric and adult participants, respectively. When the PF Plus or FA Plus bottles were unavailable, PF or SA bottles were used, respectively. Target blood volumes were 4 mL for pediatric bottles and 10 mL for adult bottles. Samples were assessed for volume adequacy defined as ±20% of the target volume based on bottle weights recorded before and after inoculation. Inoculated aerobic blood culture bottles were incubated for 5 days and mycobacterial blood cultures for 42 days. Pathogens detected by blood culture were ranked by frequency. A case of typhoid fever was defined as a participant with a blood culture positive for *Salmonella* Typhi, identified using API 20E biochemical test system (bioMerieux, Marcy l’Etoile, France). As ratio of *Salmonella* Typhi BSI to *Escherichia coli* BSI may be a surrogate for typhoid incidence [19], we included this ratio in our description of BSI for each study period. In-hospital mortality was recorded for inpatient cases and for outpatient cases determination of vital status was attempted via a 4–6 week follow-up visit.

Antimicrobial susceptibility testing was performed by disk diffusion according to the standards of the Clinical and Laboratory Standards Institute (CLSI, Wayne, PA, USA). Susceptibility interpretations were based on the 2019 CLSI guidelines and interpretive criteria, classified as susceptible, intermediate, or resistant [20]. Drugs tested included ampicillin, chloramphenicol, trimethoprim-sulfamethoxazole, ciprofloxacin, and ceftriaxone. Susceptibility testing for azithromycin was not peformed. Multi-drug resistant (MDR) *Salmonella* Typhi was defined as resistance to ampicillin, chloramphenicol, and trimethoprim-sulfamethoxazole.

#### Global positioning system coordinates and mapping

Study staff collected participant residence global positioning system (GPS) coordinates at the village level in 2007–08 and at the household level in subsequent studies using a hand-held Garmin eTrex GPS (Garmin Ltd., Olathe, KS, USA). For participants for whom study staff were unable to collect coordinates, self-reported village names were entered into the Tanzania National Addressing and Postcode System (NAPS) and coordinates for the calculated center of the village were used [21]. The GPS coordinates from the household level were presented at the village level using the calculated center of the village as the waypoint. GPS of the residence locations for cases were plotted at the village level using Quantum Geographic Information System (QGIS, v2.18.7). Population density was used *in lieu* of urban or rural classification. Population densities were calculated using 2012 census data for population by village divided by village area [22]. Comparative population density was then expressed by dividing the calculated densities into quartiles.

#### Health care utilization surveys

Healthcare utilization survey (HCUS) studies were conducted in the catchment area of KCMC and MRRH in 2011 and 2018. As described in prior publications, the 2011 HCUS was done in June through July of 2011 in Moshi Urban and Moshi Rural Districts where the respective populations at the time were estimated to be 184,292 and 466,732 [23,24]. The survey was designed to elucidate healthcare-seeking behaviors. The survey included general questions, such as what type of facility respondents would elect to receive medical care. More specific questions about preferred healthcare locales were also asked including, ‘to which facility would you go if you were unwell with a fever lasting ≥3 days?’ Heads of household only included responses to questions regarding age categories for whom they had a household member.

As previously described [25], the 2018 HCUS study was performed in Moshi Urban (population 201,150), Moshi Rural (population 509,431), and expanded to include Hai District (population 229,971). The 2018 HCUS was repeated in both the long rain and dry season, but only dry season responses were used in this analysis as it more closely matched the time of year the 2011 HCUS was performed. In contrast to the 2011 HCUS, the 2018 survey solicited responses for males and for females, respectively, within each age group.

### Adjustments for case under-ascertainment

Incidence for the catchment area was calculated using the absolute number of cases identified by hospital-based fever surveillance and then adjusting based upon factors that would contribute to under-ascertainment of cases. These adjustments are termed ‘multipliers,’ as they are the multiplicative inverse of the relevant proportions. For example, a time multiplier used to account for weekends, during which enrollment was inactive, would be calculated as the number of days in a week divided by the number of enrollment days (i.e., 7/5 = a multiplier of 1.4 to adjust for gaps in surveillance). As the 2011 HCUS only surveyed Moshi Urban and Moshi Rural Districts, only cases from those two Districts were included in incidence calculations. The 2016–18 incidence calculations included typhoid cases from Moshi Urban, Moshi Rural and Hai District. Multipliers derived from 2011 HCUS data were used for the 2007–08 and 2011–14 incidence estimates whereas multipliers derived using 2018 HCUS data were used for 2016–18 incidence estimates.

The *hospital multiplier* was incorporated to account for healthcare-seeking preferences reported in the HCUS. This multiplier takes into account which type of healthcare facility survey respondents are likely to seek and, in the event of choosing a hospital, at which specific hospital they would choose to seek care. In this way, it aims to account for cases missed due to care-seeking at facilities not under surveillance. We used individual level responses to the question ‘to which facility would you go if you were unwell with a fever lasting ≥ 3 days?’ to derive our hospital multiplier. Ranking one of our sentinel facilities as either a first or second choice constituted an affirmative answer to be included in the multiplier calculation. The *enrollment multiplier* accounted for patients who were screened as being eligible for the study but did not enroll in the hospital-based surveillance for any reason. The *blood drawn multiplier* accounts for patients for whom blood draw was attempted, but blood for culture was not obtained. The *study duration multiplie*r was used to adapt the different length studies into an annual incidence report. The *diagnostic sensitivity multiplier* was employed to account for the sensitivity of blood culture for *Salmonella* Typhi. A sensitivity of 61% (95% Confidence Interval [CI] 52–70%) was utilized based on the results reported in a systematic review of the sensitivity of blood culture for *Salmonella* Typhi [26]. The *time multiplier* accounts for the fact that fever surveillance enrollment only took place Monday through Friday. As each hospital multiplier was derived to estimate the incidence at each sentinel facility independently, when surveillance took place at both facilities, the total number of cases adjusted for blood culture sensitivity was averaged. Moderate to high burden was defined as incidence ≥10 per 100,000 whereas low incidence was defined as <10 per 100,000 [1].

### Statistical analysis and Bayesian hierarchical incidence model

Data were entered into an Access database (Microsoft, Redmond, WA, USA) using the Cardiff Teleform data capture system (initially Cardiff, Vista, CA initially but later OpenText, Waterloo, Ontario, Canada). The incidence calculations, described in the following paragraph, were conducted in R version 4.0.2 using the rijags package version 4–10. All other analyses were performed using STATA 15.1 (Stata Corp, College Station, TX, USA). We assessed for a difference in the number of MDR isolates and prior antibacterial use by period using a test for trend on a chi-squared distribution.

The standard multiplier method or hybrid surveillance approach would involve direct computation of incidence estimates based on multiplying observed incidence estimates by the multiplier adjustments described above. However, this approach does not account for the uncertainty in the multiplier adjustments themselves. We extend the standard method by incorporating these adjustments as parameters into a Bayesian hierarchical incidence model with suitable priors. A detailed description of the data, parameters and overall specification of this model are provided in the Supplementary Appendix (**S1 Text**). This includes **Table A in S1 Text** which summarizes the sources and notation for the observed data; and it includes **Table B in S1 Text**, a description and notation for the unknown parameters in the model. The modeling framework, including assumptions, distributions, prior and posterior probabilities are likewise detailed.

Incidence risk estimates and 95% credible intervals (CrI) from the Bayesian hierarchical model were stratified into three age categories: <5, ≥ 5 through <15, and ≥15 years, based on age distribution reports supplied by the Tanzanian National Bureau of Statistics (NBS) [27]. Population reports and age distributions from the 2002 and 2012 census were averaged for 2007–08 age-specific population estimates [22,28]. Data from the 2012 census was used directly for the 2011–14 period. Populations used for 2016–18 incidence calculations utilized 2012 census age distributions and NBS projections based on 2012 census data [11]. In order to describe how typhoid fever incidence estimates derived from hybrid surveillance might vary by hypothetical febrile illness scenario, we performed a one-way sensitivity analysis of our incidence estimates using responses to an alternate question on the HCUS with an unspecified duration of fever: ‘to which healthcare facility would you go for fever?’ Risk ratios of overall annual incidence of typhoid fever were generated for pair-wise comparison of each surveillance period.

## Results

### Hospital-based fever surveillance

**Fig 1:**
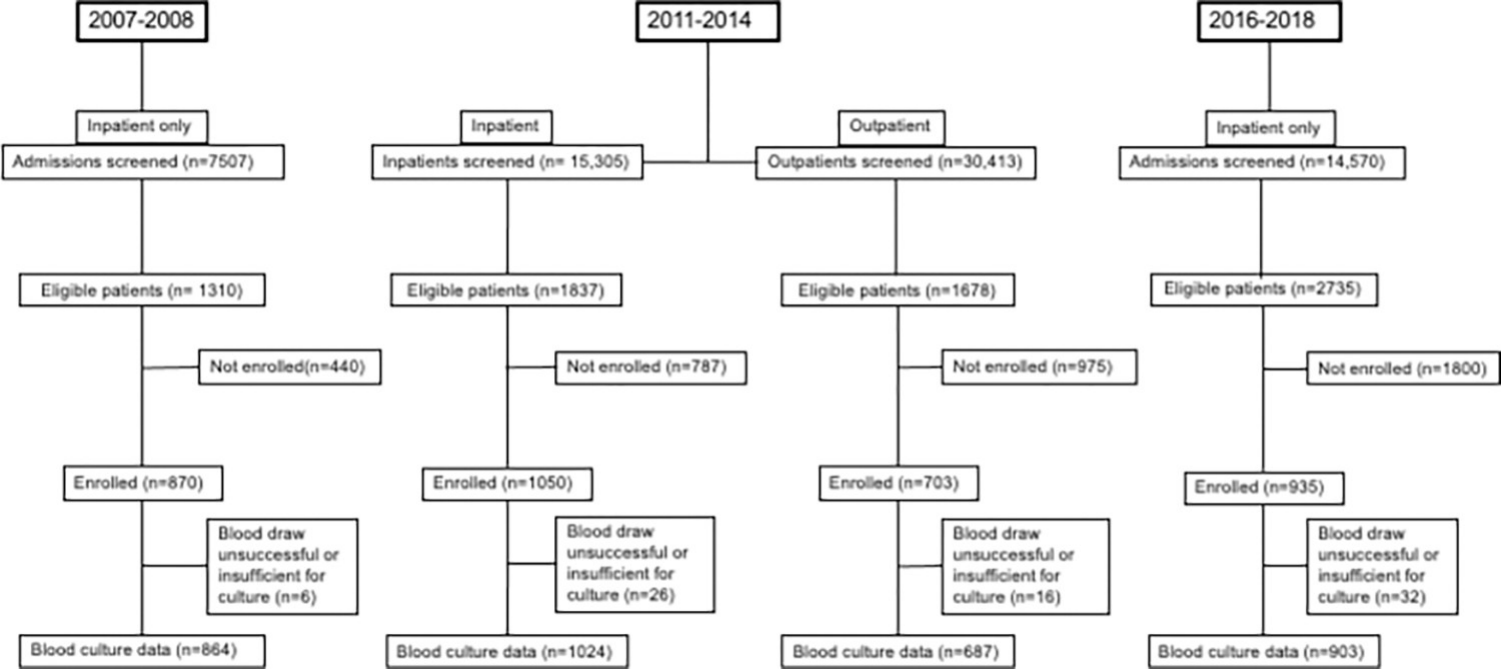
Screening and enrollment flow diagram for patients seeking care at Kilimanjaro Christian Medical Centre and Mawenzi Regional Referral Hospital in Moshi, Tanzania, 2007–2018.

A total of 3,558 participants (43.0%) of 8,266 eligible patients were enrolled in our fever surveillance studies; 870 in 2007–08, 1753 in 2011–14, and 935 in 2016–18 (**Fig 1**). Of participants, 1,338 (37.6%) were aged <5 years, 328 (9.2%) were from 5 through 14 years, and 1,892 (53.2%) were from those aged ≥15 years. Blood culture was collected for 3,465 (97.4%) of participants, 703 (20.3%) of whom were outpatients. Adequate blood culture volume was collected for 695 (44.7%) of 1,555 pediatric and 1,677 (87.8%) of 1,910 adult participants, and the adequacy in each study is shown in **Table C in S1 Text**. The three most common causes of bloodstream infection (BSI) for each of the study periods are ranked in **Table 1**. *Salmonella* Typhi was ranked as number one across the collective study periods, accounting for 50 (15.1%) of 331 pathogens isolated. However, *Salmonella* Typhi dropped in causal rank from the number one cause of BSI in the 2007–08 study period to second most common cause in the 2011–2014 study period, and then to a multi-way tie for the third-ranked cause of BSI in 2016–18 (**Table 1**). The ratio of *Salmonella* Typhi BSI to *E*. *coli* BSI was 2.3, 0.8 and 0.3 across the three study periods. During each study period respectively, 171 (42.9%) of 399, 685 (39.1%) of 1752, and 416 (45.8%) of 909 participants reported receiving antibacterials prior to enrollment (p = 0.004).

**Table 1:** Causes of bloodstream infection by rank order, Kilimanjaro Region, Tanzania 2007–2018.

Study period	Rank Order	Pathogen	Number of Isolates	(%)
2007–2008				
	1	*Salmonella enterica* serovar Typhi	32	(23.0)
	2	*Streptococcus pneumoniae*	14	(10.1)
	3	*Escherichia coli*	11	(7.9)
		Total Isolates	139	
2012–2014				
	1	*Escherichia coli*	19	(16.8)
	2	*Salmonella enterica* serovar Typhi	15	(13.3)
	3	*Streptococcus pneumoniae*	7	(6.2)
		Total Isolates	113	
2016–2018				
	1	*Escherichia coli*	11	(13.9)
	2	*Staphylococcus aureus*	7	(8.9)
	3	*Salmonella enterica* serovar Typhi*	3	(3.8)
		Total Isolates	79	
Overall				
	1	*Salmonella enterica* serovar Typhi	50	(15.1)
	2	*Escherichia coli*	41	(12.4)
	3	*Streptococcus pneumoniae*	24	(7.3)
		Total Isolates	331	

*Three-way tie for third most common isolated pathogen in the 2016–2018 period. The other two isolates were *Streptococcus pneumoniae* and *Cryptococcus neoformans*.

**Fig 2:**
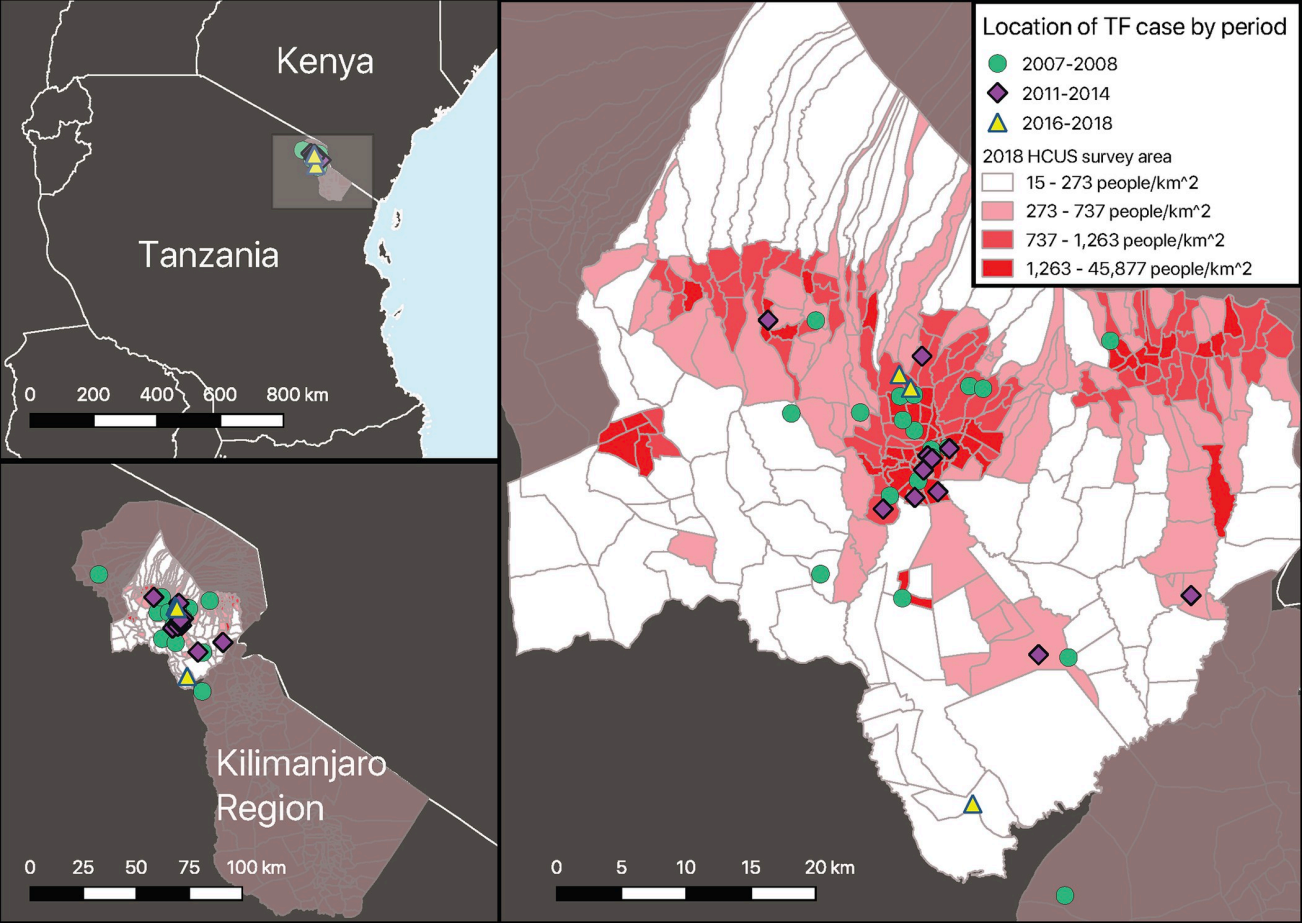
Geo-spatial location of typhoid fever cases, Kilimanjaro Region, Tanzania, 2007–2018. Map A shows Kilimanjaro Region in northern Tanzania; the region shown in map B is highlighted by the grey rectangle. Map B shows Kilimanjaro Region and, superimposed onto it, the 2018 HCUS catchment area, which included Moshi Urban, Moshi Rural and Hai districts. Map C includes village locations for typhoid cases by study period. Population density quartiles are layered on the HCUS map to allow for comparison and show potential clustering of typhoid cases in urban areas as well as cases that resided in less densely populated areas. Abbreviations: TF, typhoid fever.

In total, 50 typhoid fever cases were detected (**Fig A in S1 Text**), of which 4 (8.0%) were <5 years of age, and 10 (20.0%) were from 5 to 14 years age groups. Of all cases, 4 (8.0%) were outpatient participants, 26 (52.0%) were male, and the median (range) age was 22 (<1–60) years (**Table 2**). Prior antimalarial use was reported by 35 (70.0%) of 50 cases and prior antibacterial use by 20 (40.0%) of 50 cases. There were two typhoid fever cases who were HIV-infected in the 2007–08 study period and none in subsequent study periods. The geographic distribution of case residence is shown in **Fig 2**. The proportion of *Salmonella* Typhi isolates that were MDR (**Table 3**) was 8 (26.7%) of 30 in 2007–08, 6 (42.9%) of 14 in 2011–14, and 2 (66.7%) of 3 in 2016–18 (test for trend p = 0.1). Among inpatients, no typhoid fever case died in hospital. Among the 6 outpatient typhoid fever cases, 4 were confirmed alive at 4–6 week follow-up, while 2 had unknown vital status.

**Table 2:** Demographic and clinical characteristics of typhoid fever cases, Kilimanjaro Region, Tanzania, 2007–2018.

Variables	2007–2008		2011–2014		2016–2018		Total	
	(n = 32)		(n = 15)		(n = 3)		(n = 50)	
**Demographic characteristics**								
Age, n (%)								
<5 years	2	(6.3)	1	(6.7)	1	(33.3)	4	(8.0)
5–14 years	5	(15.6)	5	(33.3)	0	(0)	10	(20.0)
≥15 years	25	(78.1)	9	(60.0)	2	(66.7)	36	(72.0)
Gender, n (%)								
Male	17	(53.1)	8	(53.3)	1	(33.3)	26	(52.0)
Female	15	(46.9)	7	(46.7)	2	(66.7)	24	(48.0)
**Medications** *								
Prior antimalarials, n (%)	24	(80.0)	9	(60.0)	2	(100.0)	35	(74.5)
Prior antibacterials, n (%)	12	(46.2)	6	(40.0)	2	(66.7)	20	(45.5)
**Admission history and findings** *								
Illness duration,** days, median (range)	14	(1–30)	7	(1–30)	7	(3–7)	10	(1–30)
Abdomen tender to palpation, n (%)	5	(25.0)	4	(26.7)	0	(0)	9	(20.9)

*proportions reflect total number of responses to relevant question

**at time of enrollment

**Table 3:** Antimicrobial resistance of *Salmonella* Typhi Isolates, Kilimanjaro Region, Tanzania, 2007–2018.

	2007–2008		2011–2014		2016–2018	
Antibacterials	*R	† (%)	*R	(%)	*R	(%)
Ampicillin	28	(90.3)	12	(85.7)	2	(66.7)
Chloramphenicol	6	(20.0)	6	(42.9)	3	(100.0)
Trimethoprim Sulfamethoxazole	27	(90.0)	12	(85.7)	3	(100.0)
Multi Drug Resistance	8	(26.7)	6	(42.9)	2	(66.7)
Nalidixic Acid	0	(0)	1	(7.1)	0	(0)
Ciprofloxacin^‡^	0	(0)	0	(0)	1	(33.3)
Ceftriaxone^‡^	0	(0)	0	(0)	0	(0)

* R, the number of resistant isolates

† Proportions based on the total number of isolates tested

‡1 isolate was intermediate to ceftriaxone from 2007–2008

§32 isolates were intermediate for ciprofloxacin, 19 from 2007–2008 and 13 from 2011–2014

### Health care utilization survey results

In 2011 a total of 810 households were sampled, comprising 3,089 household members. Of all household members, 225 (7.3%) were <5 years of age, 655 (21.2%) were ≥5 and <15 years, and 2,209 (71.5%) were ≥15 years of age. In 2018 a total of 718 households were sampled, comprising 2,744 household members. Of all household members, 282 (10.3%) were <5 years of age, 525 (19.1%) were ≥5 and <15 years, and 1937 (70.6%) were ≥15 years of age.

### Incidence risk estimates of typhoid fever

**Fig 3:**
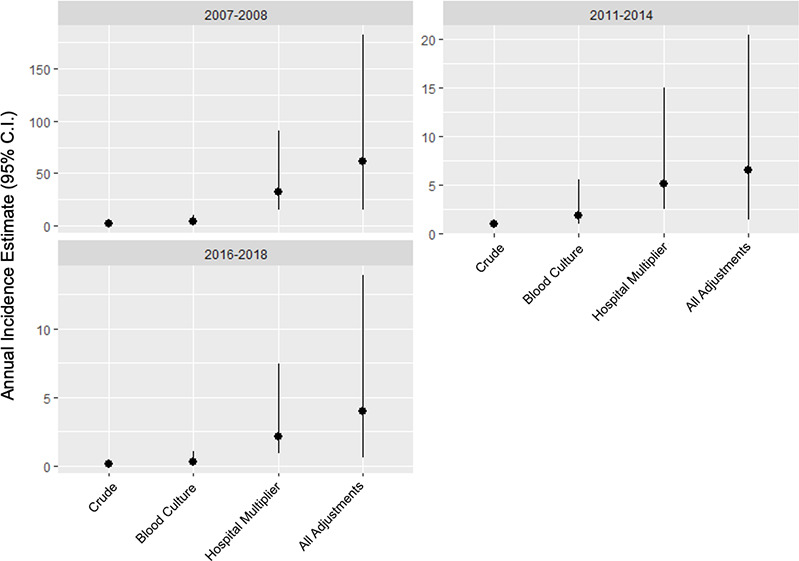
Crude incidence and key adjustments of incidence estimates using hybrid surveillance and Bayesian hierarchical model, Kilimanjaro Region, Tanzania, 2007–2018. The graphs depict the application of the multiplier method and Bayesian estimation model for each study period in order to show the adjustments from an initial crude incidence to the final incidence point estimates (per 100,000 persons) and to show the credible intervals around the point estimates at each stage of adjustment. The crude incidence is first adjusted to account for the imperfect sensitivity of Blood Culture; this adjusted incidence is further adjusted by the hospital multiplier to account for case under-ascertainment by sentinel site surveillance. The far right of each plot’s x-axis, All Adjustments, reflects the full model as described in Methods and Supplementary Methods.

The number of typhoid cases who resided within the study catchment areas for the 2011 and 2018 HCUS surveys were 23 (71.9%) of 32, 12 (80.0%) of 15, and 3 (100.0%) of 3 across the three study periods, respectively, making a total of 38 (76.0%) of the 50 cases residing within catchment areas. Adjustments using multipliers to account for under-ascertainment by our sentinel surveillance, imperfect sensitivity of blood culture, and eligible patients who did not participate are shown in **Table D in S1 Text**. Parameterizing the adjustment multipliers into the Bayesian hierarchical model, annual typhoid fever incidence risk was estimated as 61.5 (95% CrI 14.9–181.9), 6.5 (95% CrI 1.4–20.4), and 4.0 (95% CrI 0.6–13.9) per 100,000 persons in 2007–08, 2011–14, and 2016–18, respectively (**Table 4 and Fig 3**). Incidence estimates by conventional multiplier methods, without integration into a Bayesian hierarchical model, are shown in **Table E in S1 Text** Trace plots and posterior density plots for annual case estimates are shown in **Fig B in S1 Text** Corresponding Gelman-Rubin statistics are shown in **Table F in S1 Text** Sensitivity analysis comparing hospital multipliers derived from responses to the alternative hypothetical febrile illness question ‘to which healthcare facility would you go for fever?’ is shown in **Table 5**. Using the hospital multiplier derived from this question about healthcare seeking for fever of unspecified duration, point incidence was estimated as 148.9 (95% CrI 63.7–319.1), 15.4 (95% CrI 6.0–38.5), and 8.1 (95% CrI 1.0–28.5) in 2007–08, 2011–14, and 2016–18, respectively. The risk ratios for each surveillance are shown via pairwise comparisons in **Table 6**.

**Table 4:** Typhoid fever incidence estimates, Kilimanjaro Region, Tanzania, 2007–2018.

Age group (years)	KCMC crude cases*	KCMC adjusted cases**	MRRH inpatient cases*	MRRH outpatient cases*	MRRH adjusted cases**	Estimated annual cases***	Estimated Population	Annual incidence per 100,000 (95% CrI)
2007–2008								
Age <5	1	21.1 (8.3–72.3)	N/A	NA	0 (0–0)	46.3 (1.0–193.1)	72,663	63.7 (1.4–265.8)
Age 5–14	2	111.7 (47.1–317.5)	1	NA	4.8 (2.5–16.5)	123.4 (15.6–399.1)	195,442	63.1 (8.0–204.2)
Age ≥ 15	3	111.6 (57.7–291.0)	16	NA	71.5 (41.1–165.8)	198.0 (72.3–495.6)	329,994	60.0 (21.9–150.2)
Overall						367.7 (88.9–1087.8)	598,099	61.5 (14.9–181.9)
2011–2014								
Age <5	N/A	N/A	1	0	5.7 (2.6–20.8)	7.6 (0.2–32.6)	70,807	10.8 (0.2–46.1)
Age 5–14	0	0.03 (0–3.9)	2	3	25.7 (13.0–71.3)	16.4 (4.1–48.0)	155,528	10.5 (2.6–30.9)
Age ≥ 15	0	0.02 (0–1.8)	5	1	28.7 (15.1–79.4)	18.2 (5.1–52.3)	424,694	4.3 (1.2–12.3)
Overall						42.2 (9.4–132.9)	651,029	6.5 (1.4–20.4)
2016–2018								
Age <5	1	22.3 (8.6–81.3)	0	NA	0 (0–0.5)	22.9 (0.5–98.8)	110,017	20.8 (0.4–89.8)
Age 5–14	0	0.01 (0–1.2)	0	NA	0 (0–0.4)	0.04 (0–2.1)	355,438	0.01 (0–0.6)
Age ≥ 15	0	0.01 (0–1.2)	2	NA	14.3 (7.1–46.1)	14.9 (1.4–53.1)	474,858	3.1 (0.3–11.2)
Overall						37.9 (5.4–132.6)	940,312	4.0 (0.6–13.9)

*Crude cases restricted to the HCUS catchment area (n = 38)

** Sentinel facility adjusted cases have been adjusted for blood culture sensitivity and healthcare facility preferences

***Cases adjusted for blood culture sensitivity, healthcare facility preference, blood drawn, enrollment, Monday-Friday enrollment, study duration, and total number of surveillance facilities. Application of Bayesian methods to estimate incidence and credible intervals (CrI) are provided in Methods and Supplementary Methods.

Abbreviations: y, years; KCMC, Kilimanjaro Christian Medical Centre; MRRH, Mawenzi Regional Referral Hospital

**Table 5:** Sensitivity analysis for overall typhoid incidence estimates, Kilimanjaro Region, Tanzania, 2007–2018.

	KCMC crude cases	KCMC adjusted cases	MRRH inpatient cases	MRRH outpatient cases	MRRH adjusted cases	Estimated annual cases	Estimated Population	Annual incidence per 100,000* (95% CrI)
To which healthcare facility would you go if you were unwell with a fever lasting ≥3 days?								
2007–2008	6	244.6 (129.0–595.5)	17	NA	76.2 (43.9–179.8)	367.7 (88.9–1087.8)	598,099	61.5 (14.9–181.9)
2011–2014	0	0.04 (0–2.8)	8	4	61.5 (32.0–162.7)	42.2 (9.4–132.9)	651,029	6.5 (1.4–20.4)
2016–2018	1	22.2 (8.6–80.9)	2	NA	14.4 (7.1–46.2)	37.9 (5.4–132.6)	940,312	4.0 (0.6–13.9)
To which healthcare facility would you go if you were unwell with fever?								
2007–2008	6	659.6 (349.5–1391.3)	17	NA	230.3 (146.6–460.6)	890.7 (380.8–1908.3)	598,099	148.9 (63.7–319.1)
2011–2014	0	0.1 (0–8.2)	8	4	160.0 (89.7–384.7)	100.5 (39.0–250.4)	651,029	15.4 (6.0–38.5)
2016–2018	1	56.7 (18.8–196.5)	2	NA	20.7 (10.4–64.1)	75.9 (9.6–258.3)	940,312	8.1 (1.0–28.5)

*Incidences listed are “overall incidences” and a combination of point estimates across all age groups

Abbreviations: KCMC, Kilimanjaro Christian Medical Centre; MRRH, Mawenzi Regional Referral Hospital; CrI, credible intervals

**Table 6:** Typhoid fever incidence risk ratio pair-wise comparisons for each surveillance period, Kilimanjaro Region, Tanzania, 2007–2018.

Surveillance Periods Compared	Risk ratio (95% CrI)
**To which healthcare facility would you go if you were unwell with a fever lasting ≥3 days?**	
2007–2008 vs. 2011–2014	11.4 (4.3–25.1)
2007–2008 vs. 2016–2018	25.8 (4.6–93.9)
2011–2014 vs. 2016–2018	2.5 (0.4–8.9)
**To which healthcare facility would you go if you were unwell with fever?**	
2007–2008 vs. 2011–2014	11.7 (4.5–26.2)
2007–2008 vs. 2016–2018	39.5 (5.8–152.2)
2011–2014 vs. 2016–2018	3.7 (0.5–14.0)

Abbreviations: CrI, credible intervals

## Discussion

We estimated moderate typhoid incidence in 2007–08 and low incidence during later surveillance periods, but with overlapping credible intervals across study periods. While our data could be in line with global reports that suggest decreasing incidence of typhoid fever worldwide [29], it is important to note that reports from South Africa, India, and Malawi [4,30–33] showed endemic typhoid incidence varied substantially between years of surveillance, including incidence increases after several years of low incidence. While there is no accepted explanation for this variation, it could reflect fluctuating herd immunity, introduction of new strains into communities, or changes in water quality and sanitation [29,34]. In the context of these reports, rather than interpreting our three surveillance periods as successive decreases in incidence, the higher typhoid incidence in 2007–08 could represent an incidence spike followed by lower endemic incidence observed in the subsequent surveillance periods. Given the overlapping credible intervals around our point estimates and the evidence elsewhere of high variation in annual typhoid incidence, we would not conclude from our data that typhoid incidence is declining in Kilimanjaro, Tanzania. Rather we would conclude that we did not observe any signal of increasing incidence since the initial surveillance in 2007–08, and our findings highlight the need for longitudinal bloodstream infection surveillance systems in typhoid endemic countries.

Active, population-based surveillance is the most accurate and thorough way to determine disease incidence, but the resources required render it unfeasible in many typhoid-endemic settings. Multiplier methods are a well described and widely used epidemiologic approach that combines hospital-based surveillance studies with community-based healthcare utilization assessments to create incidence estimates using fewer resources than active surveillance [7,9,10]. The Typhoid Surveillance in Africa Project (TSAP) used multiplier models to create typhoid fever incidence estimates at 13 sites in sub-Saharan Africa and demonstrated the large amount of geographic variation in overall typhoid incidence, ranging from 0 (95% CI 0–0) in Sudan to 383 (95% CI 274–535) per 100,000 person years of observation in Burkina Faso [3].

Our typhoid fever incidence estimate is lower than those calculated in TSAP for Moshi, Tanzania. TSAP reported an adjusted incidence of 168 and 20 per 100,000 person years of observation in Moshi Urban District and Moshi Rural District, respectively in the 2011–14 period [3]. Part of this discrepancy is attributable to differences in multiplier derivation and methodology. We analyzed the area as one catchment and did not divide it into a separate analysis of Moshi Urban and Moshi Rural Districts. Furthermore, the multiplier in TSAP was based on healthcare seeking preferences for fever <3 days [8]. We found a median duration of illness >3 days for typhoid cases across all periods, including among outpatients, supporting our decision to use healthcare-seeking responses to fever ≥3 days for derivation of our multipliers. Our sensitivity analysis (**Table 5**) demonstrates that in hybrid surveillance that uses hypothetical healthcare-seeking scenarios, different incidence estimates can be obtained depending upon the hypothetical fever scenario used. Compared to responses for fever of ≥3 days, when asked about fever of unspecified duration, survey respondents were more likely to report that they would wait to seek care or start by seeking care at a local dispensary and less likely to report healthcare seeking at one of our sentinel facilities. This lower hypothetical report of presentation to the sentinel facilities translates to applying a larger hospital multiplier to the cases captured by our surveillance (i.e., a larger adjustment for case under-ascertainment by sentinel surveillance); and therefore a higher typhoid fever incidence estimate is calculated when fever of unknown duration is used as the healthcare seeking scenario. Using either the traditional multiplier method or Bayesian hierarchical incidence modeling, the hospital multiplier was the dominant driver of uncertainty (**Fig 3 and Table E in S1 Text**). It is therefore reasonable to conclude that uncertainty may be reduced by performing HCUS prior to starting surveillance and only surveilling facilities where most patients seek care.

*Salmonella* Typhi was the leading cause of BSI in the Kilimanjaro Region across our three fever surveillance studies, comprising 50 (15%) of 331 pathogens isolated. However, *Salmonella* Typhi dropped in causal rank from the number one cause of BSI in the 2007–08 study period to a multi-way tie for the third-ranked cause of BSI in 2016–18 (**Table 1**). Given the variation in *Salmonella* Typhi over time, it can be helpful to present this prevalence data in comparison with the prevalence of other BSI pathogens [19]. In doing so it is important to choose a pathogen with high prevalence and without an available vaccine, such as *E*. *coli*. *E*. *coli* rose from the second-ranked isolate during the 2007–08 study period to the first-ranked isolate in the latter two studies; and the smaller ratio value of S*almonella* Typhi BSI prevalence to *E*. *coli* BSI prevalence tracked with the lower typhoid incidence estimates, consistent with a recent meta-analysis of this prevalence ratio as a surrogate for typhoid incidence [19]. Typhoid fever cases were evenly distributed by sex and the majority of our cases, 36 (72.0%) of 50, were persons 15 years of age or older (Table 2). The majority of cases resided in communities that were more population dense, though there were also cases that resided in rural communities (**Fig 2**). While we cannot exclude that these cases contracted typhoid fever via exposure to an urban environment, this finding is consistent with other reports demonstrating that typhoid fever affects rural populations in Africa [35].

Antimicrobial resistance is a growing global concern, attributable in part to the frequent use of antibacterials in the community [36–38]. In our study, only 20 (40.0%) of 50 typhoid cases reported antibacterial use prior to seeking care. This was lower than the 62.2% prior antibacterial use that was reported in a 2015 study examining community antibacterial use in northern Uganda [39], and higher than the 35.2% prior antimicrobial use reported in a 2018 typhoid surveillance study in Nepal, which is closer to the epicenter of extensively drug resistant *Salmonella* Typhi [40]. In sub-Saharan Africa, penicillins and sulfonamides are among the most utilized antibacterial classes at the community level [37,41]. We observed a statistically non-significant trend in the proportion of *Salmonella* Typhi that were MDR, from approximately one third in 2007–08 to two thirds in 2016–18, although the total number of isolates was small in the 2016–18 period (**Table 3**). This is consistent with systematic review findings that the median proportion of MDR Salmonella Typhi in Africa has continued to increase each decade for the last three decades [38]. In the face of MDR *Salmonella* Typhi, fluoroquinolones, extended-spectrum cephalosporins, and azithromycin have been used as alternative treatment options for typhoid, but resistance has become increasingly common for these agents as well [42,43]. Only one of our isolates, from the 2016–18 study, was resistant to ciprofloxacin and no isolates were resistant to ceftriaxone. However, there was a high proportion of isolates with intermediate susceptibility to ciprofloxacin, 19 (61.3%) of 31 in 2007–08 and 13 (92.9%) of 14 in 2011–14.

Previous studies have estimated the average case fatality ratio (CFR) for typhoid fever at around 1% [44,45], with variation depending on geographic region, outpatient versus inpatient status, and differences in time to appropriate therapy. We observed no deaths among our typhoid cases. This lower than anticipated CFR is consistent with recent findings from a study performed in 2010 in Bangladesh where the CFR was 0.3% [46]. It is important to note that CFR is likely to be lower in the context of clinical surveillance research with routine blood culture than in routine clinical care in setting, as cases detected by fever surveillance blood cultures might be more likely to receive appropriate antimicrobials in a timely manner.

We present multi-year surveillance data spanning more than a decade in a region within sub-Saharan Africa, an area where there are very few longitudinal reports of incidence. A strength of our study is the consistency in surveillance study design and the study population, which allows for direct comparison across distinct time periods. Another strength of our study is the Bayesian hierarchical model employed to estimate incidence and to calculate uncertainty, which more thoroughly demonstrates how uncertainty is compounded at each stage of adjustment in hybrid surveillance incidence studies. We believe this approach to measuring the uncertainty around hybrid surveillance incidence estimates is a methodologic advance for disease surveillance research. A limitation of our data is that while similar enough to be compared, it comes from three separate, non-continuous study periods with slightly different eligibility criteria for febrile illness. We were unable to compare the observed incidence trends with UNICEF reported water, sanitation, and hygiene interventions [47], as there was no region-specific data available for the Kilimanjaro Region. In order to utilize a multiplier model, we assumed that the inpatient population at our sentinel facilities and the population surveyed for the HCUS were similar enough that they would have the same preferences for healthcare. While multiplier studies are an accepted method for estimating incidence, the low overall number of crude cases detected by our surveillance platform makes it difficult to distinguish random error from significant trends. Lastly, past studies have shown that a substantial portion of typhoid fever cases are diagnosed in the outpatient setting [7], so not including outpatient surveillance in the 2007–08 and 2016–18 studies may have resulted in an under-estimation of incidence.

In summary, we identified variation in typhoid incidence, but with overlapping credible intervals, in Kilimanjaro Region, Tanzania over an 11-year period. Multiplier studies are a practical and useful epidemiologic approach to estimate incidence and to inform public health policy, especially in resource-limited settings. However, there is great variation on how these multiplier studies are performed and which factors they take into account [2]. To improve comparability of surveillance results from different settings, collective refinements and methodologic standardization are needed for disease estimation using hybrid surveillance. Due to the variation in incidence in locations with endemic typhoid, policy decisions to implement typhoid conjugate vaccine and evaluation of real world vaccine efficacy would be best served by multi-year typhoid fever surveillance.

## Supporting information

S1 Text Supplementary Methods and Results. Bayesian hierarchical incidence model for hybrid surveillance. **Table A** Sources and notation for the observed data in the hierarchical Bayesian incidence model. **Table B** Description and notation for the unknown parameters in the hierarchical Bayesian incidence model. **Table C** Blood culture volume adequacy by study period. **Table D** Adjustment multipliers for typhoid fever incidence estimates, Kilimanjaro Region, Tanzania, 2007–2018. **Table E** Typhoid fever incidence estimates, Kilimanjaro Region, Tanzania, 2007–2018 by application of standard hybrid surveillance multiplier method. **Table F** Gelman-Rubin statistic estimates, and upper 95% confidence interval estimate, for each estimate of the annual number of typhoid cases from the Bayesian incidence model. **Fig A** Chronological presentation of typhoid fever cases by month, Kilimanjaro Region, 2007–2018. **Fig B** Trace plots and posterior density plots for each estimate of the annual number of typhoid cases from the Bayesian incidence model.
(DOCX)
