# Supplementary material for: Facility-based disease surveillance and Bayesian hierarchical modeling to estimate endemic typhoid fever incidence, Kilimanjaro Region, Tanzania, 2007–2018
Source: PLoS Negl Trop Dis. 2022 Jul 5;16(7):e0010516. doi: 10.1371/journal.pntd.0010516 (PMC9286265; doi:10.1371/journal.pntd.0010516)
Supplement: S1 Text — Bayesian hierarchical incidence model for hybrid surveillance. Table A Sources and notation for the observed data in the hierarchical Bayesian incidence model. Table B Description and notation for the unknown parameters in the hierarchical Bayesian incidence model. Table C Blood culture volume adequacy by study period. Table D Adjustment multipliers for typhoid fever incidence estimates, Kilimanjaro Region, Tanzania, 2007–2018. Table E Typhoid fever incidence estimates, Kilimanjaro Region, Tanzania, 2007–2018 by application of standard hybrid surveillance multiplier method. Table F Gelman-Rubin statistic estimates, and upper 95% confidence interval estimate, for each estimate of the annual number of typhoid cases from the Bayesian incidence model. Fig A Chronological presentation of typhoid fever cases by month, Kilimanjaro Region, 2007–2018. Fig B Trace plots and posterior density plots for each estimate of the annual number of typhoid cases from the Bayesian incidence model. (DOCX) [file pntd.0010516.s001.docx]

**Supplementary File**

**Supplementary Methods**

***Bayesian hierarchical incidence model for hybrid surveillance***

Hybrid surveillance, involving the pairing of facility-based surveillance with population-based healthcare utilization surveys, has been employed to generate population incidence estimates for a number of infectious diseases including typhoid. Leveraging data from multiple existing sources is less resource intensive than conducting cohort studies. The typical approach for hybrid surveillance is to adjust crude incidence estimates with several multiplicative adjustment factors to account for various factors, such as facility coverage, enrollment capture, and the sensitivity of diagnostic tests [1, 2]. We refer to this approach as the “multiplier method”.

The multiplier method has several limitations. Most importantly, it is deterministic and thus does not account for uncertainty in the calculation of the adjustment factors. Further, it is not straightforward to incorporate individual or community level risk factors that may be important in understanding heterogeneity in incidence within or between populations. In light of these limitations, we propose a Bayesian hierarchical incidence model for hybrid surveillance. For simplicity, the model we propose here does not include individual or community level risk factors, but we note as a strength of this approach that it can be extended to do so.

In this section, we first introduce our notation and describe what data we are using, then explain the parameters of interest and the prior distributions we are assuming, and finally provide the overall specification of the model.

*Observed Data and Notation*

The data used in our model comes from two sources: hospital-based fever surveillance studies and community-based healthcare utilization surveys (described above). See **Table A** for a description of the variables derived from each data source and the relevant notation.

**Table A.** Sources and notation for the observed data in the hierarchical Bayesian incidence model.

| **Data Source** | **Description** | **Notation** |
| --- | --- | --- |
| Hospital-based surveillance | Number of eligible patients at hospital *j* in surveillance study *k* | $L_{\cdot jk}$ |
|  | Number of enrolled patients in age stratum *i* at hospital *j* in surveillance study *k* | $E_{ijk}$ |
|  | Number of enrolled patients with blood drawn in age stratum *i* at hospital *j* in surveillance study *k* | $B_{ijk}$ |
|  | Number of positive typhoid cases in age stratum *i* at hospital *j* in surveillance study *k* | $T_{ijk}$ |
| Community-based healthcare utilization survey | Number surveyed in age stratum *i* from catchment area for hospital *j* in surveillance study *k* | $S_{ijk}$ |
|  | Number willing to seek care at hospital *j* if feeling unwell with a fever lasting ≥ 3 days, in age stratum *i* and surveillance study *k* | $W_{ijk}$ |

From the hospital-based surveillance studies, for each surveillance study (*k*) at each hospital (*j*) we have a number of eligible patients ($L_{\cdot jk})$ out of which only a certain number are enrolled ($E_{ijk}).$ Note that the enrolled patients are stratified by age (*i*), but not the eligible patients since we did not collect data on patients who were eligible but not enrolled. Once enrolled, a certain number of patients have their blood drawn for testing ($B_{ijk}$), out of which a certain number will be positive for typhoid ($T_{ijk}$).

In the community-based healthcare utilization surveys, we surveyed a number of individuals ($S_{ijk}$) in age stratum *i* from the catchment area for hospital *j* in surveillance study *k*. The survey question of interest was whether or not they would seek care at hospital *j* if they were feeling unwell with a fever lasting at least 3 days; the number responding positively in age stratum *i* and surveillance study *k* are denoted $W_{ijk}$.

Note that we have used aggregated data at each level of our model (i.e., the number of individuals meeting the criteria for that level—for example, all individuals in a particular age stratum-- out of the number surveyed at that level). It is straightforward to extend the model to accommodate individual-level data and adjust for measured covariates at the individual level. For simplicity and ease of comparison with the published multiplier method we use the aggregated outcomes.

*Model Parameters*

The unknown parameters in our model and relevant priors are given in **Table B.** In all cases, a conjugate prior is used: Beta priors for binomial probabilities (using the standard characterization with two positive real shape parameters) and a Gamma prior for the Poisson incidence risk (using the standard characterization with one positive real shape parameter and one positive real rate parameter).

The probability of seeking care at hospital *j* for patients in age stratum *i* in surveillance study *k* is denoted by $\gamma_{ijk}$, while the probability of a patient being enrolled at hospital *j* in surveillance study *k* is denoted by $\delta_{\cdot jk}$. Note that, as mentioned above, since we did not gather age information on eligible but not enrolled patients, the probability of being enrolled is constant across age strata. Note too that this is not a marginal probability, but the probability of being enrolled conditional on having sought care and been eligible for enrollment (for simplicity we omit the conditioning from our notation). Similarly, the probability of a patient in age stratum *i* having a blood draw sufficient to inoculate a blood culture bottle at hospital *j* in surveillance study *k* ($\varepsilon_{ijk}$) is conditional on that patient being enrolled, and again we omit the conditioning from our notation for simplicity. All three of these probability parameters are given a “flat” $Beta(1,1)$ prior; that is, the prior assigns equal weight to the entire probability space from 0 to 1, meaning we are imparting no prior belief as to where in the probability space these parameters lie.

The sensitivity of the blood culture (i.e., the probability of a patient having a positive blood culture result given the patient is a true typhoid case) is denoted by $\pi$. The hyperparameters for the prior on sensitivity were derived based the results of a systematic review of the sensitivity of blood culture for *Salmonella* Typhi [3]. The systematic review reported a sensitivity of 61% (95% confidence interval: 52%-70%), a Beta distribution with hyperparameters of $\alpha=2.550369$ and $\beta=1.630564$ approximates this central tendency and spread.

The population incidence risk of typhoid in age stratum *i* at hospital *j* in surveillance study *k* is denoted by $\lambda_{ijk}$. This risk is given the standard “vague” $Gamma(0.001,0.001)$ prior. This prior is vague because it contributes almost no information to the posterior distribution; thus, our results will be driven primarily by the observed data likelihood.

Finally, a constant study duration multiplier is applied to adjust for differences in duration of the surveillance studies. The study durations are 11.5 months for the 2007-2008 period, 32 months for 2011-2014 period, and 24 months for the 2016-2018 period; $c_{k}$ is equal to 12 divided by the duration for each study. This annualizes the incidence risk estimates derived from each study so that they are comparable. Note that this model can be extended to explicitly account for time, and thus any secular trends in incidence over the course of the studies; but for simplicity and ease of comparison with the published multiplier method we use crude estimates of annual incidence.

**Table B.** Description and notation for the unknown parameters in the hierarchical Bayesian incidence model.

| **Parameter** | **Notation** | **Prior** | **Notes** |
| --- | --- | --- | --- |
| Probability of seeking care in age stratum *i* at hospital *j* in surveillance study *k* | $\gamma_{ijk}$ | $Beta(1,1)$ | Flat prior |
| Probability of being enrolled in age stratum *i* at hospital *j* in surveillance study *k* | $\delta_{ijk}$ | $Beta(1,1)$ | Flat prior |
| Probability of having blood drawn in age stratum *i* at hospital *j* in surveillance study *k* | $\varepsilon_{ijk}$ | $Beta(1,1)$ | Flat prior |
| Sensitivity of blood culture | $\pi$ | $Beta(2.550369,1.630564)$ | Hyperparameters for prior derived from systematic review |
| Population incidence risk of typhoid in age stratum *i* at hospital *j* in surveillance study *k* | $\lambda_{ijk}$ | $Gamma(0.001,0.001)$ | Vague prior |
| Study duration multiplier | $c_{k}$ | n/a | Treated as constant |

*Model Specification*

To justify our modeling framework, we begin with a simplified motivating example. Say the true underlying incidence risk of disease, $D,$was distributed according to a Poisson distribution with parameter $\theta$, i.e.:

$$D \sim Poisson(\theta)$$

However, due to incomplete detection, we only observe some proportion of these true cases, $Y$. We can model this process as:

$$Y|D \sim Binomial(p,D)$$

where $p$ denotes the probability of detecting a case. Due to the relationship between the binomial and Poisson distributions, whereby the binomial distribution with “large” n and “small” p tends in limit to the Poisson distribution with rate parameter $\theta p$, we can model our observed cases as:

$$Y|D \sim Poisson\left( \theta p \right) ,$$

where we note that the approximation using the Poisson is reasonable in our case due to the small risk of typhoid in the study population.

The posterior distribution of the parameters is then:

$$P\left( \theta,p | Y,D \right)=\frac{P\left( Y | \theta,p,D \right)P\left( \theta\right)P(p)}{P(Y|D)} ,$$

where $P\left( Y | \theta,p \right)$ denotes the observed data likelihood from a Poisson distribution, $P(\theta)$ and $P(p)$ denote prior distributions assigned to the parameters, and $P(Y)$ is a normalizing constant.

Building from this simplified motivating example, we “chain” together multiple binomial processes to our model of interest that relates our observed case count to the underlying incidence risk.

Thus, we model the number of observed typhoid cases using the following Poisson distribution:

$$T_{ijk} \sim Poisson\left( \frac{7}{5}c_{k}\lambda_{ijk}\gamma_{ijk}\delta_{\cdot jk}\varepsilon_{ijk}\pi\right),$$

where we relate the probability parameters $\gamma,\delta,$ and $\varepsilon$ to our observed data using:

$$W_{ijk} \sim Binomial(\gamma_{ijk},S_{ijk})$$

$$E_{\cdot jk} \sim Binomial(\delta_{\cdot jk},L_{\cdot jk})$$

$B_{ijk} \sim Binomial(\varepsilon_{ijk},E_{ijk})$,

where all quantities are defined using the notation given in **Table A** and **Table B**. The $\frac{7}{5}$ constant is to adjust for the fact that fever surveillance enrollment only took place on weekdays. That is, we are assuming that if enrollment had taken place on weekends it would have occurred at a similar rate as on weekdays, and adjust our incidence estimates to take into account the missing enrollment days.

Note that we make three important assumptions for our model to be identifiable: 1) independence of the parameters, e.g. the probability of an individual seeking care ($\gamma)$ is independent of the population incidence risk ($\lambda$); 2) independence of our data sources, i.e. the hospital-based surveillance and the community-based healthcare utilization survey; and 3) the number of individuals surveyed $S_{ijk}$ in the healthcare utilization survey is treated as a fixed, not random, quantity.

The joint posterior distribution of our parameters given the data can then be written as:

$$P\left( \lambda_{ijk},\pi,\varepsilon_{ijk},\delta_{\cdot jk},\gamma_{ijk}|T_{ijk},B_{ijk},E_{ijk},L_{\cdot jk},W_{ijk} \right)=\frac{P\left( T_{ijk},B_{ijk},E_{ijk},L_{\cdot jk},W_{ijk} | \lambda_{ijk},\pi,\varepsilon_{ijk},\delta_{\cdot jk},\gamma_{ijk} \right)P\left( \lambda_{ijk} \right)P\left( \pi\right)P\left( \varepsilon_{ijk} \right)P\left( \delta_{\cdot jk} \right)P(\gamma_{ijk})}{P(T_{ijk},B_{ijk},E_{ijk},L_{\cdot jk},W_{ijk})} ,$$

where the numerator on the right-hand side consists of the likelihood term, i.e. P(data|parameters), multiplied by the prior distribution for each parameter as given in **Table B.** The denominator is simply a normalizing constant. We note that the key parameter of interest is the overall probability of incidence of typhoid and, as noted above, it is derived as the product of the relevant conditional probabilities to obtain the overall risk of typhoid.

*Estimation*

The posterior distribution for the model described above was estimated using Markov Chain Monte Carlo (MCMC) sampling with 3 parallel chains, 1000 iterations for adaptation, 100000 posterior samples, and a thinning interval of 1. The analysis was conducted in R version 4.0.2 with the rjags package version 4-10 [4]. In order to describe how typhoid fever incidence estimates derived from hybrid surveillance might vary by hypothetical febrile illness scenario, we performed a sensitivity analysis of our incidence estimates by fitting two different versions of the model with different estimates of care-seeking probability based on two different questions from the healthcare utilization survey: “To which healthcare facility would you go if you were unwell with a fever lasting ≥ 3 days?” vs. “To which healthcare facility would you go if you were unwell with a fever?”.

**Supplementary Results**

2011-2014

2016-2018

**Fig A: Chronological presentation of typhoid fever cases by month, Kilimanjaro Region, 2007-2018.** Hospital surveillance was inactive between September 1, 2008 through September 25, 2011 and June 1, 2014 through September 5, 2016.


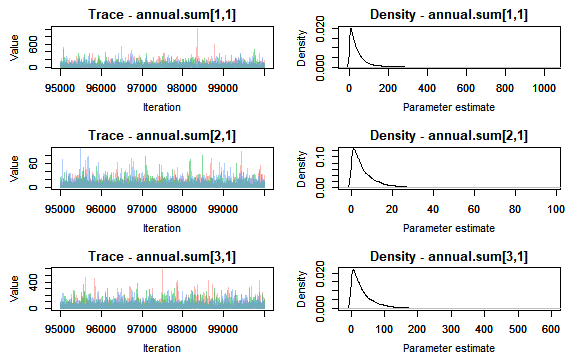

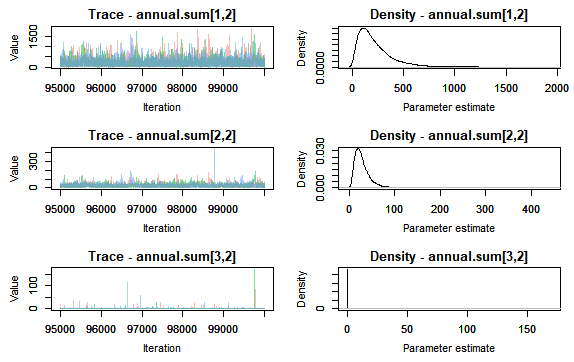


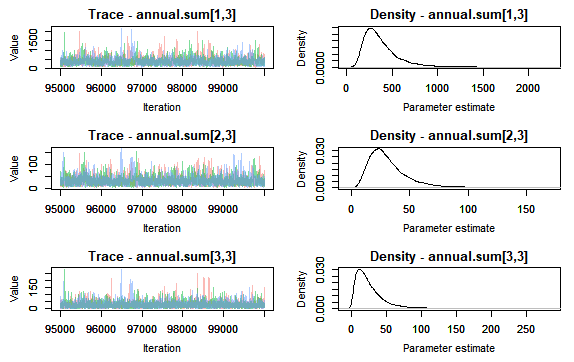


**Fig B: Trace plots and posterior density plots for each estimate of the annual number of typhoid cases from the Bayesian incidence model**. The estimates are indexed by survey period and age group, respectively (e.g. annual.sum[1,1] denotes the estimate for the annual number of typhoid cases in 2007-2008 for age <5, annual.sum[1,2] denotes the estimate for the annual number of typhoid cases in 2011-2014 for age <5, etc.).

**Table C: Blood culture volume adequacy by study period**

| Period | Age Category | | # Volume Adequate | (%) | | n* |
| --- | --- | --- | --- | --- | --- | --- |
| 2007-2008 | Pediatric | 137 | | (29.7) | 462 | |
|  | Adult | 364 | | (90.5) | 402 | |
| 2012-2014 | Pediatric | 394 | | (53.9) | 731 | |
|  | Adult | 841 | | (87.0) | 967 | |
| 2016-2018 | Pediatric | 164 | | (45.3) | 362 | |
|  | Adult | 472 | | (87.2) | 541 | |

*n= total number of blood culture samples collected

**Table D: Adjustment multipliers for typhoid fever incidence estimates, Kilimanjaro Region, Tanzania, 2007-2018**

| Multiplier Type | Multiplier equation | Multiplier for period | | | | | |
| --- | --- | --- | --- | --- | --- | --- | --- |
| **Data from health care utilization surveys 2007-2008 2011-2014 2016-2018** | | | | | | | |
| KCMC multiplier* | # of individuals interviewed / # of individuals seeking care at KCMC for fever ≥3 days |  |  |  |  |  |  |
| age <5 y |  | 225/19 | **11.84** | 225/19 | **11.84** | 282/24 | **11.75** |
| age 5-14 y |  | 655/20 | **32.75** | 655/20 | **32.75** | 525/23 | **15.91** |
| age ≥15 y |  | 2,209/100 | **22.09** | 2,209/100 | **22.09** | 1,937/129 | **15.02** |
| MRRH multiplier* | # of individuals interviewed/ # of individuals seeking care at MRRH for fever ≥3 days |  |  |  |  |  |  |
| age <5 y |  | 225/74 | **3.04** | 225/74 | **3.04** | 282/65 | **4.34** |
| age 5-14 y |  | 655/237 | **2.76** | 655/237 | **2.76** | 525/142 | **3.70** |
| age ≥15 y |  | 2,209/856 | **2.58** | 2,209/856 | **2.58** | 1,937/517 | **3.75** |
| **Data from fever surveillance studies** | | | | | | | |
| Enrollment multiplier | # of eligible patients/ # of patients enrolled in fever surveillance | 1,310/870 | **1.51** | 4,221/1,753 | **2.41** | 2,735/935 | **2.93** |
| Blood drawn multiplier | # of patients enrolled in fever surveillance/ # of patients for whom blood culture was performed |  |  |  |  |  |  |
| age <5 y |  | 388/383 | **1.01** | 614/578 | **1.06** | 336/323 | **1.04** |
| age 5-14 y |  | 86/86 | **1.00** | 169/168 | **1.01** | 73/69 | **1.06** |
| age ≥15 y |  | 396/395 | **1.00** | 970/965 | **1.01** | 526/511 | **1.03** |
| Study duration multiplier | # of months per year/study duration (in months) | 12/11.5 | **1.04** | 12/32 | **0.38** | 12/24 | **0.5** |
| Time multiplier | # of days in a week/ # of enrollment days per week | 7/5 | **1.40** | 7/5 | **1.40** | 7/5 | **1.40** |
| Diagnostic sensitivity multiplier |  | 1/0.61 | **1.64** | 1/0.61 | **1.64** | 1/0.61 | **1.64** |

*Multipliers for KCMC and MRRH are derived from the healthcare utilization surveys described in Methods.

Abbreviations: y, years; KCMC, Kilimanjaro Christian Medical Centre; MRRH, Mawenzi Regional Referral Hospital

**Table E: Typhoid fever incidence estimates, Kilimanjaro Region, Tanzania, 2007-2018 by application of standard hybrid surveillance multiplier method.**

| **Age group (years)** | **KCMC crude cases*** | **KCMC adjusted cases**** | **MRRH inpatient cases*** | | **MRRH outpatient cases*** | | **MRRH adjusted cases**** | **Estimated annual cases***** | **Estimated**  **Population** | **Annual incidence per 100,000 by standard multiplier method** | | **Annual incidence per 100,000 by Bayesian incidence model^** |
| --- | --- | --- | --- | --- | --- | --- | --- | --- | --- | --- | --- | --- |
| **2007-2008** |  |  | |  | |  |  |  |  |  | |  |
| **Age <5** | 1 | 19.4 | | N/A | | NA | 0 | 43.3 | 72,663 | | 60 | 63.7 (1.4-265.8) |
| **Age 5-14** | 2 | 107.4 | | 1 | | NA | 4.5 | 123.4 | 195,442 | | 63 | 63.1 (8.0-204.2) |
| **Age ≥ 15** | 3 | 108.6 | | 16 | | NA | 67.7 | 194.5 | 329,994 | | 59 | 60.0 (21.9-150.2) |
| **Overall** |  |  | |  | |  |  | 361.2 | 598,099 | | 60 | 61.5 (14.9-181.9) |
| **2011-2014** |  |  | |  | |  |  |  |  | |  |  |
| **Age <5** | N/A | 0 | | 1 | | 0 | 5.0 | 5.6 | 70,807 | | 8 | 10.8 (0.2-46.1) |
| **Age 5-14** | 0 | 0 | | 2 | | 3 | 22.7 | 11.9 | 155,528 | | 8 | 10.5 (2.6-30.9) |
| **Age ≥ 15** | 0 | 0 | | 5 | | 1 | 25.4 | 13.4 | 424,694 | | 3 | 4.3 (1.2-12.3) |
| **Overall** |  |  | |  | |  |  |  | 651,029 | | 5 | 6.5 (1.4-20.4) |
| **2016-2018** |  |  | |  | |  |  |  |  | |  |  |
| **Age <5** | 1 | 19.3 | | 0 | | NA | 0 | 20.8 | 110,017 | | 19 | 20.8 (0.4-89.8) |
| **Age 5-14** | 0 | 0 | | 0 | | NA | 0 | 0 | 355,438 | | 0 | 0.01 (0-0.6) |
| **Age ≥ 15** | 0 | 0 | | 2 | | NA | 12.3 | 13.0 | 474,858 | | 3 | 3.1 (0.3-11.2) |
| **Overall** |  |  | |  | |  |  |  | 940,312 | | 4 | 4 (0.6-13.9) |

^*^*Crude cases restricted to the HCUS catchment area (n=38)

** Sentinel facility adjusted cases have been adjusted for blood culture sensitivity and healthcare facility preferences

***Cases adjusted for blood culture sensitivity, healthcare facility preference, blood drawn, enrollment, Monday-Friday enrollment, study duration, and total number of surveillance facilities.

^Bayesian incidence estimates and credible intervals are shown in Table 4 in main manuscript. They are included here for ease of comparison with the standard (non-Bayesian) multiplier method.

Abbreviations: y, years; KCMC, Kilimanjaro Christian Medical Centre; MRRH, Mawenzi Regional Referral Hospital

**Table E: Gelman-Rubin statistic estimates, and upper 95% confidence interval estimate, for each estimate of the annual number of typhoid cases from the Bayesian incidence model**. The estimates are indexed by survey period and age group, respectively (e.g. annual.sum[1,1] denotes the estimate for the annual number of typhoid cases in 2007-2008 for age <5, annual.sum[1,2] denotes the estimate for the annual number of typhoid cases in 2011-2014 for age <5, etc.).

| **Parameter** | **Gelman-Rubin Statistic** | **Upper 95% C.I.** |
| --- | --- | --- |
| annual.sum[1,1] | 1.00 | 1.00 |
| annual.sum[1,2] | 1.00 | 1.00 |
| annual.sum[1,3] | 1.00 | 1.00 |
| annual.sum[2,1] | 1.00 | 1.00 |
| annual.sum[2,2] | 1.00 | 1.00 |
| annual.sum[2,3] | 1.00 | 1.00 |
| annual.sum[3,1] | 1.00 | 1.00 |
| annual.sum[3,2] | 1.07 | 1.07 |
| annual.sum[3,3] | 1.00 | 1.00 |

1. Andrews JR, Barkume C, Yu AT, Saha SK, Qamar FN, Garrett D, et al. Integrating Facility-Based Surveillance With Healthcare Utilization Surveys to Estimate Enteric Fever Incidence: Methods and Challenges. J Infect Dis. 2018;218(suppl_4):S268-s76. Epub 2018/09/06. doi: 10.1093/infdis/jiy494. PubMed PMID: 30184162; PubMed Central PMCID: PMCPMC6226762.

2. Crump JA, Youssef FG, Luby SP, Wasfy MO, Rangel JM, Taalat M, et al. Estimating the incidence of typhoid fever and other febrile illnesses in developing countries. Emerg Infect Dis. 2003;9(5):539-44. Epub 2003/05/10. doi: 10.3201/eid0905.020428. PubMed PMID: 12737736; PubMed Central PMCID: PMCPMC2972755.

3. Mogasale V, Ramani E, Mogasale VV, Park J. What proportion of Salmonella Typhi cases are detected by blood culture? A systematic literature review. Ann Clin Microbiol Antimicrob. 2016;15(1):32. Epub 2016/05/18. doi: 10.1186/s12941-016-0147-z. PubMed PMID: 27188991; PubMed Central PMCID: PMCPMC4869319.

4. Plummer, Martyn. rjags: Bayesian Graphical Models using MCMC. R package version 4-10, 2019. <https://CRAN.R-project.org/package=rjags>. Accessed 3 March 2021.
